# A Study on Over-Molded Copper-Based Flexible Electronic Circuits

**DOI:** 10.3390/mi13101751

**Published:** 2022-10-16

**Authors:** Mona Bakr, Martin Hubmann, Frederick Bossuyt, Jan Vanfleteren

**Affiliations:** 1 Center for Microsystems Technology, Ghent University, 9052 Ghent, Belgium; 2 Department of Polymer Engineering and Science, Polymer Processing, Montanuniversitaet Leoben, 8700 Leoben, Austria

**Keywords:** flexible printed circuit boards, injection molding, film insert-based technology

## Abstract

Over-molding has been proposed in recent years as an integrated functional flexible circuit board in a plastic part. This method uses the conventional process for film insert technology. Over-molding has attracted significant attention across many industries due to its potential to deliver different electrical functions in a variety of different part geometries, especially in automotive interiors and home appliances. While it has great application potential, manufacturing challenges continue throughout foil fabrication and injection molding. This raises challenges for designers and researchers responsible for maintaining the reliability of such electronic flexible circuits. Therefore, the purpose of this research paper is to improve some of the over-molding process parameters. On 0805 and 1206 over-molded zero-ohm resistors, electrical, mechanical, and failure characterization was performed. Those components were mounted in parallel, perpendicular, and 45° angled arrangements on two different polymer substrates, polyimide (PI) and polyethylene terephthalate (PET) using lead-free solder, low-melt solder, and conductive adhesive paste. Moreover, as an over-molding material, polycarbonate (PC) with medium viscosity was used. The effect of using different mold shapes (corner mold, 2 mm flat mold, and 3 mm flat mold) and injection molding process parameters (injection speeds and melt temperature) was studied.

## 1. Introduction

Plastic products are becoming very popular for mass production since they are cheap, versatile, and easy to manufacture in a variety of forms. Plastic products arefabricated using different manufacturing processes including, injection molding, thermoforming, blow molding, and extrusion. Since the 1990s, a special injection molding process based on insert film technology has gained great importance from researchers and manufacturers. This process is called the in-mold or over-molding process. It is based on a foil insert that could be an electrical foil with assembled components which is inserted into an injection mold that defines the shape of the part that is to be produced. An injection molding machine then rapidly injects the molten thermoplastic into the mold and then the temperature is controlled to quickly cool the produced part. A packing pressure is applied that pushes additional melt into the cavity during the initial stage of this cooling phase to compensate for the volumetric shrinkage of the solidifying melt. The in-mold process is greatly preferred in the automotive industry because it can offer lightweight plastic products with good mechanical strength together with functional circuits [1,2]. This elevates user expectations since this technology can generate three-dimensional structures. Therefore, it is sufficient that the electronic systems fit a convenient form factor of the shape into which they will be integrated. Many researchers worked intensively to integrate different electrical functionalities like light emitting diodes (LEDs), light guides, numerous touch switches or slides, and near field communication (NFC) antennas into plastic products using the in-mold process [3,4]. By fabricating optical touch panels, organic light emitting diodes (OLEDs), foils, and disposable healthcare sensors, T. Alajoki et al. [5] demonstrated the feasibility of hybrid in-mold integration. O. Rusanen et al. [6] developed the IMSE technology (injection-molded structural electronics) by integrating pre-formed silver-based printed electronics and standard electronics components into 3D plastics. Moreover, Juntunen et al. [7], and Kololuoma et al. [8] realized wearables. A novel concept that could serve as a platform for the integration of photovoltaic (PV) cells into plastic products was developed by M. Bakr et al. [9]. Moreover, adhesion mechanics and a demonstration of how to power LEDs wirelessly using an NFC antenna and a chip were discussed in [10]. Wimmer et al. [11] concluded that printed conductive structures must be optimized for the forming process to achieve and maintain the highest electrical conductivity possible. O. M. Tuomikoski et al. [12] demonstrated indoor air quality monitoring and red-green-blue (RGB) luminaire devices with both printed and injection-molded parts. In addition to this, some researchers have expressed interest in examining the impact of the process on different electronic packages, flexible foil materials, metallization, assembly methods, and mold shapes to determine the effect of plastic materials on microsystems [13,14,15,16,17].

The most critical factor in over-molding technology is the compatibility of the various materials to achieve sufficient adhesion between the insert foil and the polymer injected by the injection molding machine. Many research studies were conducted to determine the impact of material combination and injection molding processing parameters on the interface adhesion, which, in turn, determines the product’s properties. These experiments did not include any electronics, instead, only foils were over-molded with the injected polymer. Yamaguchi, S. [18] studied the influence of crystallization on the adhesion of polypropylene (PP) foil to PP-injected polymer at the interface. Additionally, Chen et al. examined the characterization of temperature profiles while using PET and PC foils with PC [19] and PP [20] as an injected polymer, observing that both combinations yielded satisfactory results. Moreover, other studies have defined fundamental concepts of interface adhesion using one or more of the following surface treatment mechanisms: interface interlocking, molecular diffusion and entanglement, crystallization, and adsorption theory [21,22,23,24]. However, surface treatments are not suggested for large-scale production because they increase the overall cost.

The primary purpose of this research paper is to demonstrate the use of different copper-based foils as substrate foil. In contrast to our work in [10], this paper examines the influence of various mold shapes and the influence of used component assembly materials like lead-free solder, conductive adhesive, and low-melt solder on the electrical performance after over-molding. The first section introduces the over-molding process for integrating electronic components, followed by the experimental procedures which include mold and foil design, adhesion test, process simulation, component assembly, and over-molding. Finally, the final section evaluates the shear test and electrical characterization results.

## 2. Experimental Procedures

The process flow begins with the mold design and ends with the over-molded plastic part.

### 2.1. Mold Design

**Figure 1:**
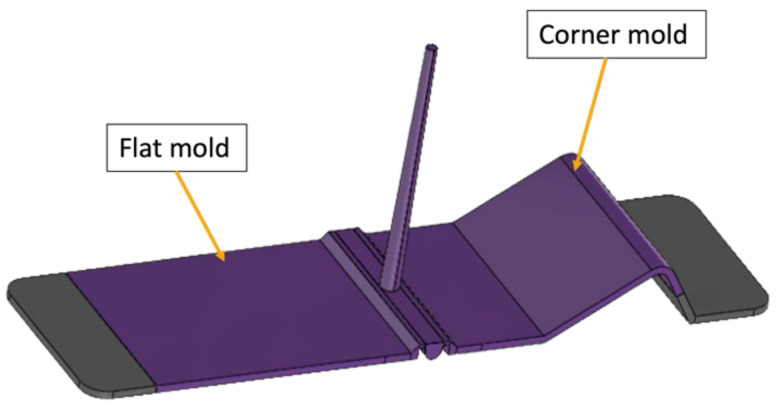
Mold design.

The mold was designed to enable the over-molding in three different cavities. A flat plate mold whose wall thickness can be adjusted to 2 or 3 mm and a corner-shaped mold with an inner radius of 4 mm and wall thickness of 3 mm as depicted in Figure 1.

### 2.2. Foil Design

**Figure 2:**
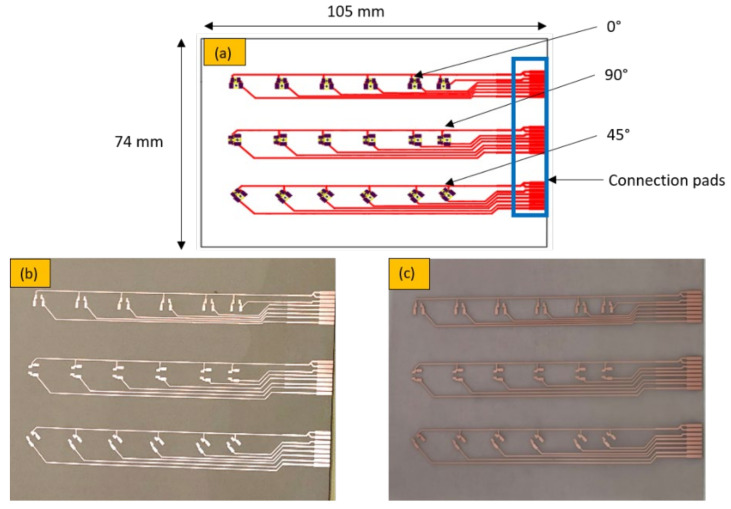
(**a**) Schematic foil design, (**b**) PI-Cu foil, and (**c**) PET-Cu foil.

According to the mold design, we designed the circuit foils to fit in the three mold cavities with dimensions 105 × 74 mm as illustrated in Figure 2, where the connection pads are properly positioned on the right side; each pad is connected to a resistor location and then to the ground. The pads are connected to 18 components and are enabled to take the measurements before and after over-molding. Additionally, it enables the assembly of 0-ohm resistors in a variety of packages (0805 and 1206) and orientations (0°, 90°, and 45°).

### 2.3. Adhesion and Peel Tests

**Figure 3:**
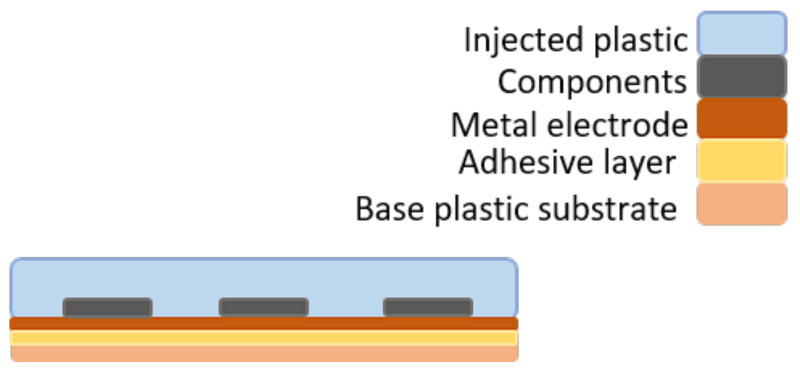
Typical materials stack.

In this paper, the medium viscosity PC Makrolon 2805 (Covestro AG, Leverkusen, Germany) [25] is used as an injection material. The amorphous and transparent PC is known for its mechanical strength as well as high heat and flame resistance [26]. A durable over-molded flexible electronic product should always be fabricated with suitable material combinations with good adhesion between the flexible circuit foil (base plastic substrate) and the over-molded material (injected plastic) as depicted in Figure 3. In this research, the adhesion performance between PC and two commercial copper cladding foils was studied. The foils were composed of three layers: a polymer layer, a copper layer, and an adhesive layer. The composition of the used foils is given in Table 1.

The peel strength between the foil adhesive layers shown in Table 1 and the PC molding material was evaluated using a 90° peel test. To that end, the copper was fully removed from some foils that were subsequently over-molded within a 2 mm plate mold with 280 °C melt temperature and injection speed of 70 cm^3^/s. Table 2 presents the over-molded samples for the peel test.

**Figure 4:**
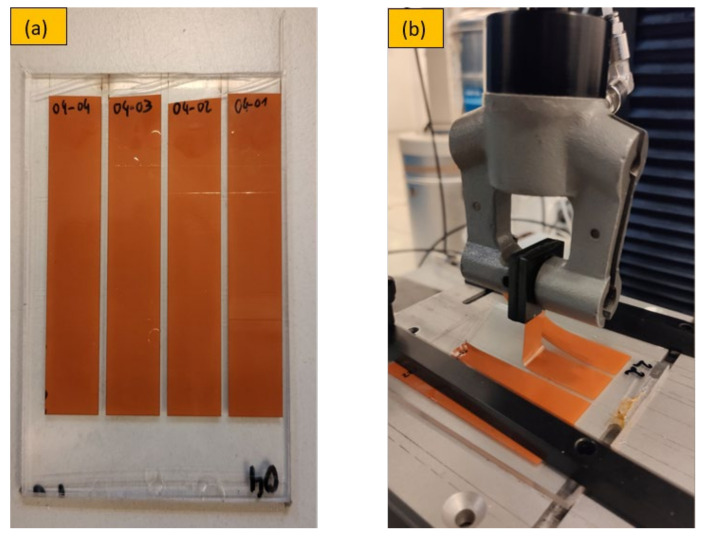
Peel-off tests setup.15 mm wide foils strips (**a**), Instron 5500R tensile test machine (**b**).

The molded foils were then laser cut into 15 mm wide foils strips as depicted in Figure 4a (n = 4) and peeled using an Instron 5500R (Illinois Tool Works Inc., Glenview, IL, US) tensile test machine with an attached peel-off fixture as shown in Figure 4b. A peel speed of 10 mm/min was chosen and static load cells were 10 N and 100 N used for PI and PET foils, respectively.

**Figure 5:**
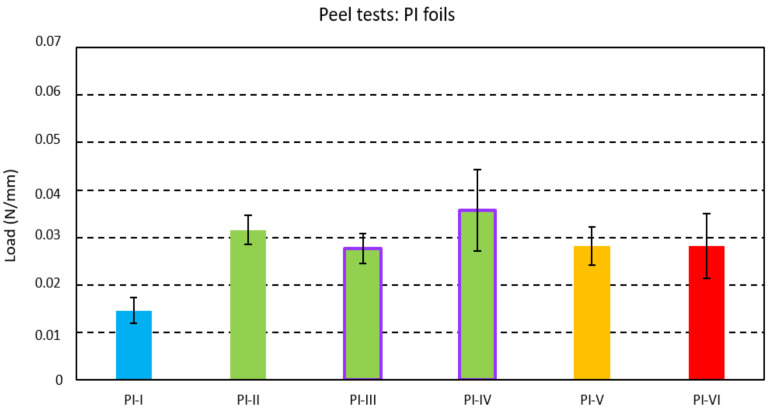
Peel strength for the PI foils with an acrylic-based adhesive layer.

**Figure 6:**
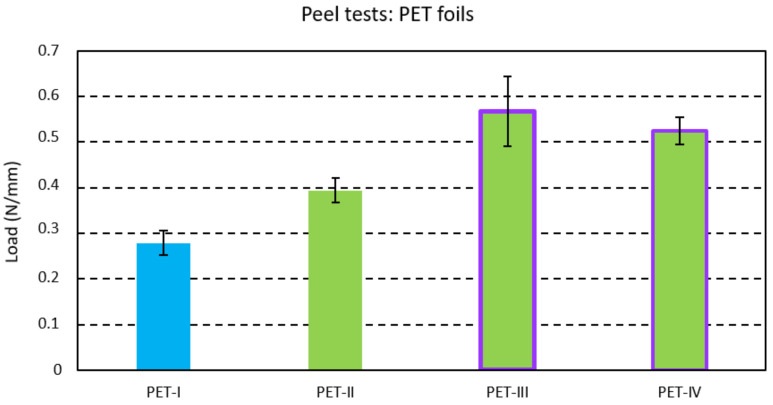
Peel strength for the PET foils with a polyurethane-based adhesive layer.

The mean peel strength was recorded for a peel length of 40 mm for each foil strip. The averaged peel strength per investigated foil with standard deviation indicating the variation between the foil strips is given in Figure 5 for PI foils with an acrylic adhesive layer and in Figure 6 for PET foils with polyurethane as an adhesive layer. The polyurethane-based adhesive in PET foils showed about 10 times larger adhesion with the injected PC compared to the acrylic-based layer in PI foils.

One-Way ANOVAs with Tukey tests (with 95% confidence interval) for the PI foils (from PI-I to P-VI) yielded a significantly lower adhesion for the foil molded at the low mold temperature (PI-I). While increasing the mold temperature from 80 °C (PI-I) to 100 °C (PI-II) increased the adhesion, a further increase to 120 °C (PI-VI) could not be confirmed to aid bonding. The long residual cooling time of 50 s (PI-II)—effectively tempering the acrylic close to its glass transition temperature [29]—could not be confirmed to aid adhesion compared to a shorter cooling time of 20 s (PI-V). No effect on the bonding could be confirmed when surface-treating the acrylic layer using corona activation (PI-III) or using oxygen plasma (PI-IV).

Likewise, One-Way ANOVAs with Tukey tests (with a 95% confidence interval) for PET foils were made. Increasing the mold temperature from 80 °C (PET-I) to 100 °C (PET-II) was again found to yield a significantly higher peel strength. A further increase could be noted when applying corona or oxygen plasma treatment to the polyurethane-based adhesive layer (PET-III and PET-IV).

### 2.4. Simulation

**Figure 7:**
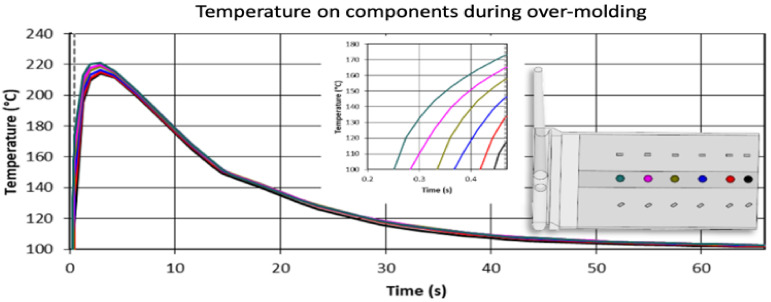
The temperature profile of the components during over-molding with PC.

The temperature profile of the resistors during the injection molding cycle was numerically investigated using the commercial injection molding simulation software Autodesk Moldflow Insight (AMI) as depicted in Figure 7. An AMI model was created exemplarily for the 2 mm plate (3,141,193 elements), comprising the eighteen 1206 components (in total 77,748 elements) molded as solid aluminum oxide blocks. The two layers of the PI films were modeled separately as acrylic and polyimide parts (188,900 elements). Moreover, the mold (3,116,641 elements) with cooling channels was included too. Figure 7 shows the temperature development on the 1206 components during over-molding with the PC when a melt temperature of 300 °C, a mold temperature of 100 °C, and an injection speed of 70 cm^3^/s is used. The grey dashed line indicates the end of filling (~0.4 s) with the inset focusing on this stage of the injection molding cycle. The highest temperatures of approximately 215 °C are reached later during packing. The used temperatures are lower than the melting temperature of the soldering material which is 260 °C. Therefore, it can be assumed that the resistors will remain fully functional and reliable after over-molding.

### 2.5. Foil Fabrication and Assembly

For all used copper-PI and copper-PET foils, the copper was patterned using lithography and etching. The foil fabrication includes the cleaning of the surface from any foreign substances and particles, photoresist coating, UV exposure and photoresist development, the etching of the exposed metal film, and removal of the photoresist. To protect the patterned copper from oxidation, the copper is treated with an organic solder preservative (OSP). Finally, laser ablation is occasionally used to cut the foil into the desired insert shape and to create an opening that acts as a gate for the polymer to flow during the over-molding process.

**Figure 8:**
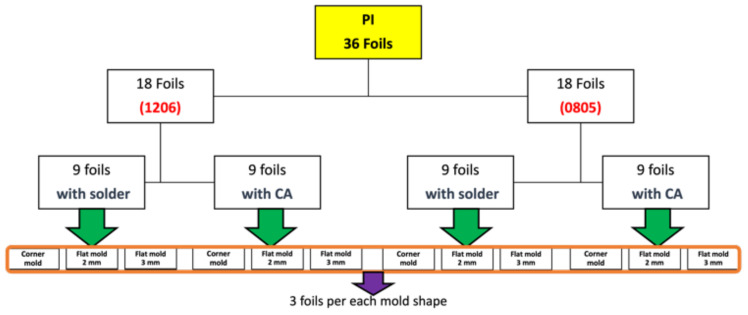
Overview of PI-Cu foils.

**Figure 9:**
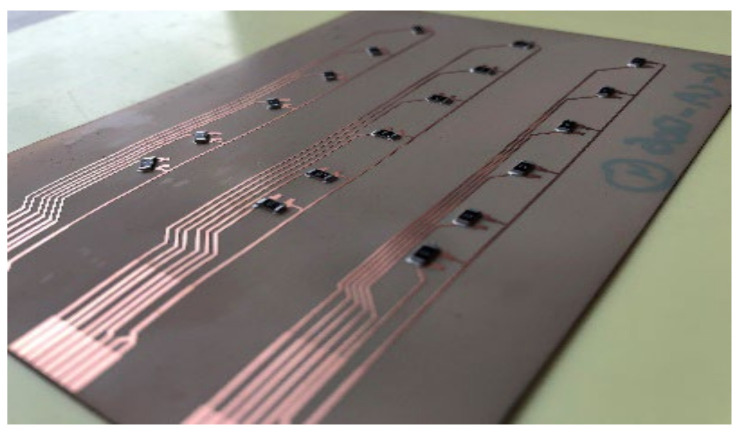
Real-life PI-Cu foil with components.

The mold is roughly 98.5 mm in length, and we ensured that components were assembled 22 mm away from the mold side wall. The assembly step involves mounting SMD resistors (zero-ohm resistors) acquired from Yageo to the PI-Cu and PET-Cu foils. The assembly plan for the PI-Cu foils included 18 foils assembled using a standard lead-free solder paste [30] and 18 foils assembled using thermoset silver-based conductive glue (Henkel CE 3103) [31]. Lead-free solder and conductive glue were applied to the copper pads of the copper traces, followed by the resistor assembly. Following that, the foils are heated until the solder particles reflow and harden in a reflow oven. On the other hand, in the case of conductive glue, it is cured for 20 min at 120 °C in a convection oven. Figure 8 depicts the used foils assembly plan for the PI-Cu foils and Figure 9 shows a real-life image of a foil with assembled components.

**Figure 10:**
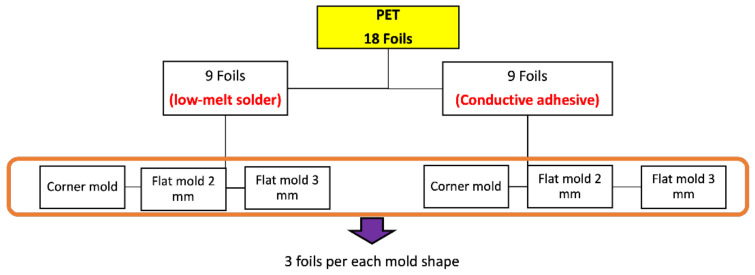
Overview of used PET-Cu foils.

**Figure 11:**
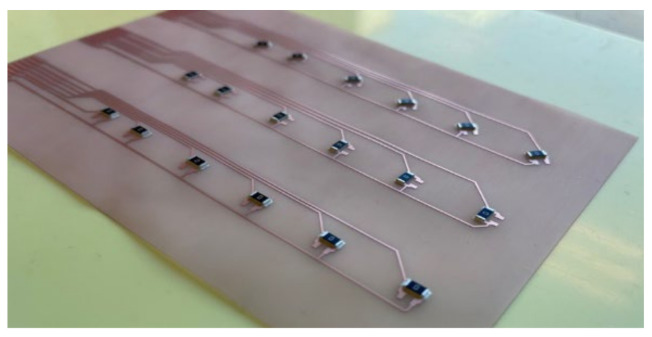
Real-life PET-Cu foil with components.

Moreover, the assembly plan for the PET-Cu foils included 9 foils assembled using a low-temperature melt solder paste (Interflux DP5600, Interflux Electronics, Gent, Belgium) [32] and 9 foils assembled using thermoset silver-based conductive glue (Henkel CE 3103). The same steps were taken for the PI-Cu foils. The only difference is the use of the reflow oven for the low-melt solder paste and low-melting temperature PET foils. The reflow oven was configured for preheating for 5 min at 160 °C, followed by 15 min of reflow at the same temperature at 160 °C, then cooling for 6 min. Figure 10 depicts the used foils assembly plan for the PET-Cu foils and Figure 11 shows a real-life image of a foil with assembled components.

### 2.6. Over-Molding

**Figure 12:**
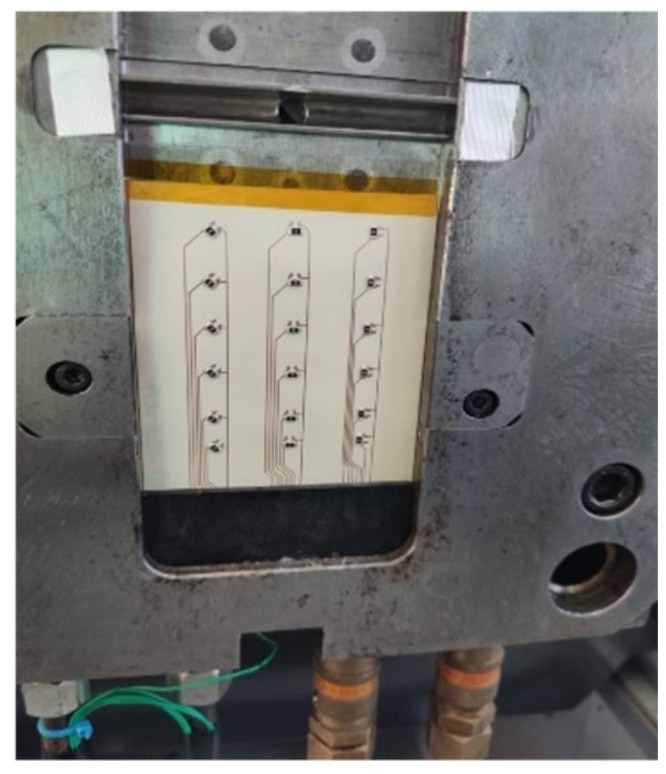
Foil fixed in a flat mold.

**Figure 13:**
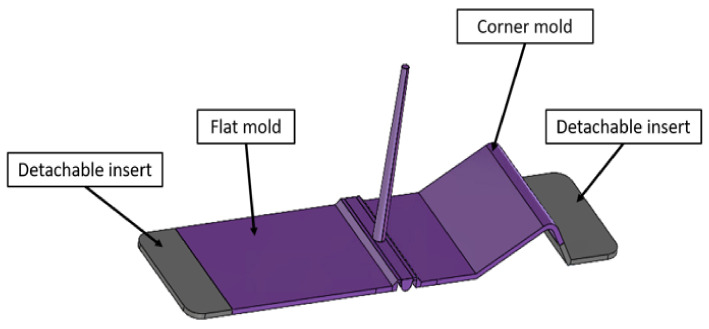
Location of the detachable inserts.

The foils were placed in the mold as indicated in Figure 12 and fixed using temperature-resistant adhesive tape. The detachable inserts were then added to keep the contacts free from being over-molded, they were located as depicted in Figure 13. As a drawback of this simple approach, the films occasionally showed wrinkles after over-molding at the transition to the insert.

An Arburg Allrounder 470A (Arburg GmbH + Co. KG, Loßburg, Germany) injection molding machine equipped with a 25 mm screw was used for performing the over-molding. The two cycles of a Wittmann Tempro plus D 160 (WITTMANN Technology GmbH, Austria) temperature control unit were used to regulate the temperatures of the two mold halves to 100 °C. This mold temperature was chosen based on the performed peel test results outlined in Section 2.3 above.

The used PC (Makrolon 2805, Coversto AG, Leverkusen, Germany) was dried for 3 h at 120 °C before molding. The packing pressure was set to 400 bar for 15 s and the residual cooling time to 50 s for all tests. The dosing volume was set either to 50 cm^3^ for the corner and the 3 mm plate or to 40 cm^3^ for the 2 mm plate. The switch-over point (velocity-to-pressure-controlled filling) was adapted for each injection speed and melt.

**Figure 14:**
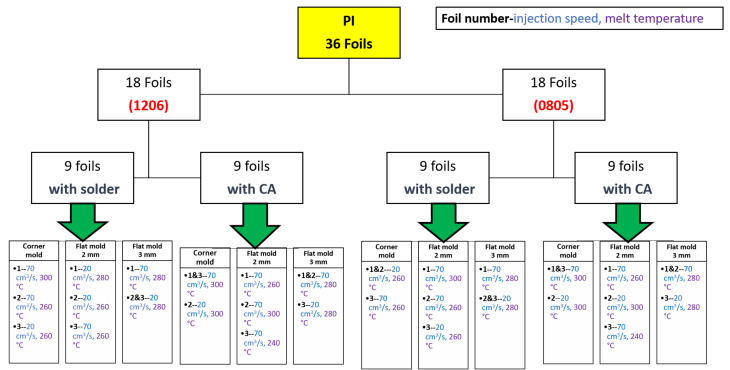
Overview of over-molded foils.

Figure 14 lists the 36 produced over-molded PI-Cu foils with their corresponding settings. Those foils yielded a broad molding window in which (visually) undamaged parts could be produced. The aim was to find those boundaries of the injection molding process by changing the melt temperature between 240, 260, 280, and 300 °C and the injection speed to either 20 or 70 cm^3^/s. In preceding tests with the PET foils (without components) it was shown that they were significantly more sensitive to the formation of wrinkles during over-molding. This issue could be reduced when selecting a higher melt temperature and faster injection speed setting of 300 °C and 70 cm^3^/s, respectively.

## 3. Results

### 3.1. Shear Test

**Figure 15:**
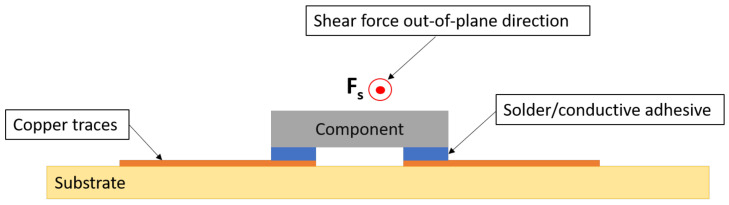
Cross-sectional view for shear off component.

The strength of the joints was assessed using shear tests resembling the DIN EN 62137 1–2 norm. The shear tests were performed on a Bruker UMT-2 (Bruker Corporation, Billerica, MA, US) mechanical tester platform with a 100 N load cell at the Polymer Competence Center Leoben GmbH (PCCL). A schematic illustration of the setup is given in Figure 15. The components were aligned in 180° orientation and sheared using a rectangular 4 mm width chisel that moved at 1/4th of the component’s height at a speed of 6 mm/min.

Table 3 displays the shear load at the break for the investigated 0805 and 1206 components assembled using solder, low-melt solder, and conductive adhesive on PI and PET foils. As a general observation, the load at break increased by increasing the component size. Moreover, higher shear loads at break were recorded when the components were soldered not glued using the conductive adhesive.

### 3.2. Foils Evaluation

**Figure 16:**
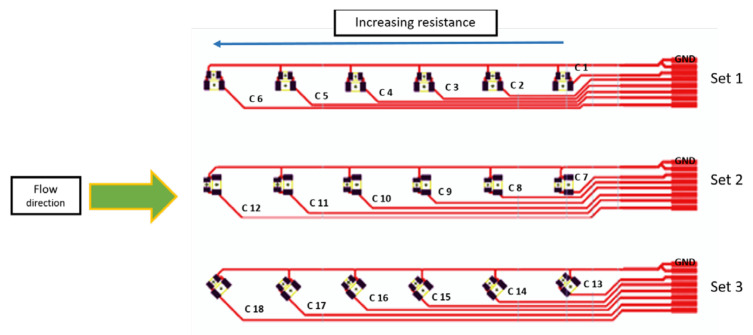
Labeling of components on the foil.

Foils tested were assembled using 0805 and 1206 SMD 0-ohm resistors. These resistors were selected in our study because they can be easily assembled manually in a reproducible way. To know the actual resistance of the resistors, we used a multimeter to check the value of ten 0 Ω resistors from both packages to act as the R*_initial_* of the component, which was 0.2 Ω before assembling the resistors on the foil. Each 0 Ω resistor mounted on the flex has a different track length (L) between the component and the connection pad as depicted in Figure 16.

The resistance of the assembled 0 Ω resistor increases proportionately to the length of the copper track. The measured resistance is the sum of the resistance of the used component, the resistance of the copper track, the resistance of the used connection (solder, low-temperature solder, and conductive adhesive), and the probe’s contact resistance. In other words:

(1) R measured=R initial+R track length+R connection+R contact

Assembly using lead-free solder proved its applicability when using PA6 as the injected molding material and PI-Cu foils as presented in our previous work in [10]. However, in this paper, assembly using low-temperature solder and conductive adhesive is also studied. Moreover, different foils, PET-Cu-based foils are evaluated to broaden the research area on the influence of over-molding parameters on electrical characterization. The following sections are divided into two main subsections, PI-Cu foils and PET-Cu foils including a detailed explanation of the measurements.

#### 3.2.1. Measurements on PI-Cu Foils

##### Measurements on Samples Assembled with Lead-Free Solder

Using lead-free solder, the resistance before over-molding was the same for the 18 components. Therefore, we can check the effect of track length on the measurements using an average value of six resistance values (*R*) from component one (C1) to component six (C6) as presented in Table 4.

To compare *R_measured_* and *R_theoretical_* we had to consider *R_initial_ =* 0.2 Ω, the ρ of copper = 1.7 × 10^−8^ mΩ, the track thickness = 35 µm, and the track width = 300 µm. We calculated R *_theoretical_* for 18 resistors using Equation (1), and Table 5 presents the data for set 1 of the resistors. No significant difference in resistance was found between the measured and calculated values. Moreover, this comparison demonstrates that the primary contributor to the measured resistance is the change in resistance of the copper track length; both solder connections and probe contacts contribute insignificantly to the measured resistance.

**Figure 17:**
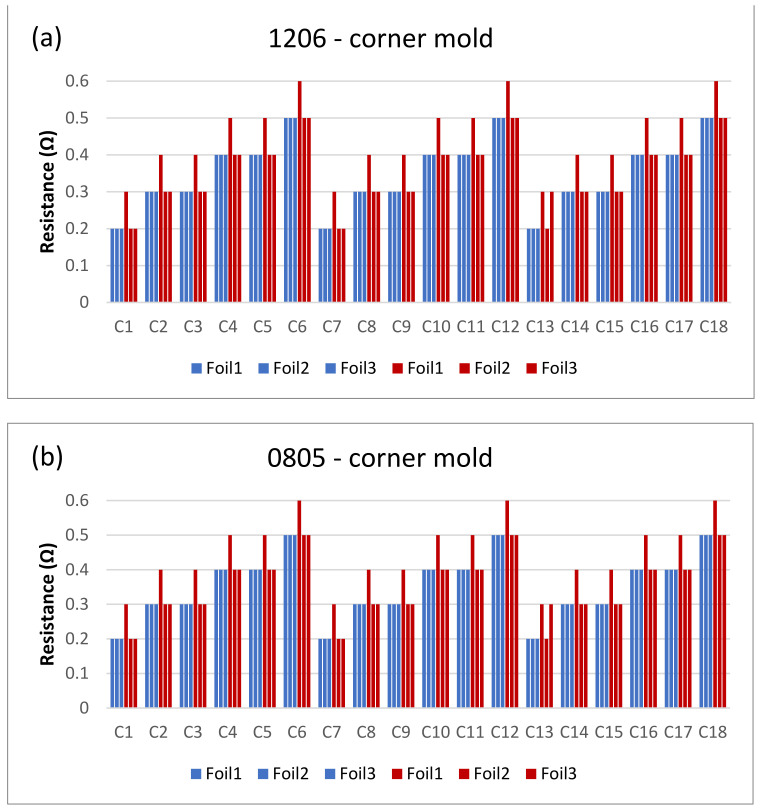
Resistance measurements for 1206 resistors (**a**) and 0805 resistors (**b**) molded in corner mold. Blue bars (before over-molding) and red bars (after over-molding).

The next step is to clamp the foils into the mold and perform an over-molding cycle. Figure 17 depicts the resistance measurements for the corner mold for the 1206 and 0805 components. The measurements were taken for three foils before (blue bars) and after (red bars) over-molding had almost the same readings except for foil 1 after over-molding which had an increase in resistance of 0.1 Ω.

**Figure 18:**
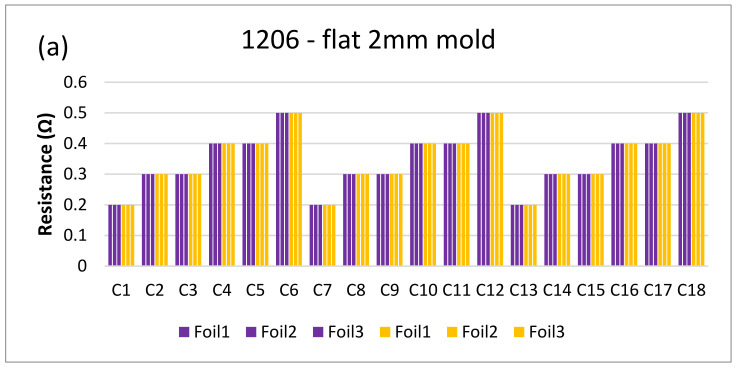

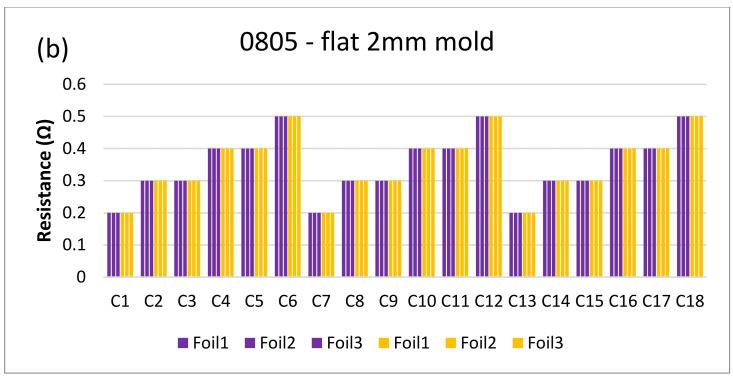
Resistance measurements for 1206 resistors (**a**) and 0805 resistors (**b**) molded in the 2 mm flat mold. Purple bars (before over-molding) and yellow bars (after over-molding).

**Figure 19:**
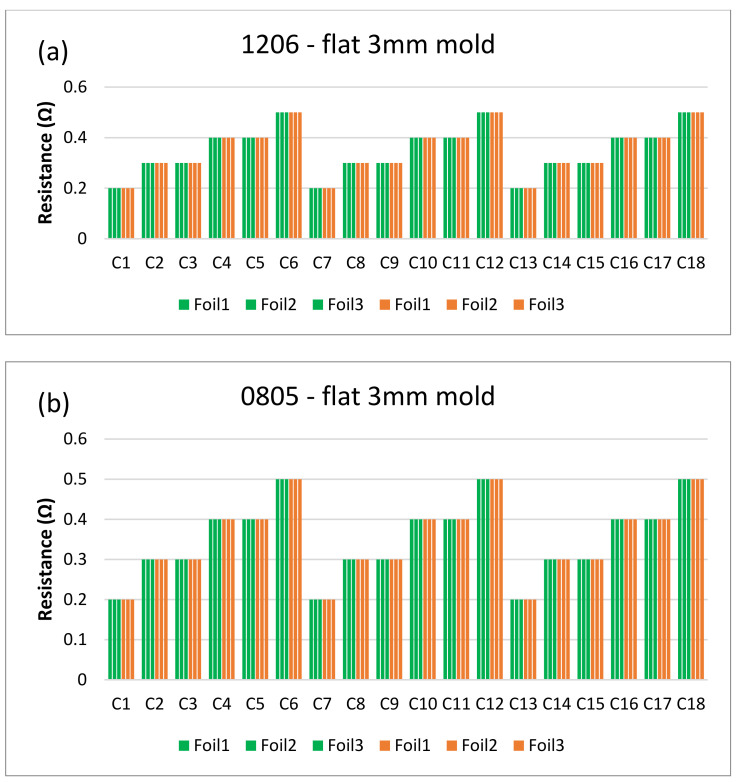
Resistance measurements for 1206 resistors (**a**) and 0805 resistors (**b**) molded in the 3 mm flat mold. Green bars (before over-molding) and orange bars (after over-molding).

Figure 18 and Figure 19 show the measurements for the three foils in the flat mold in 2-mm and 3-mm cavities, respectively. The over-molding did not affect the measured resistance, indicating that solder-based components can withstand the over-molding process and also that the internal stresses in the flat molds are lower than in the corner mold.

**Figure 20:**
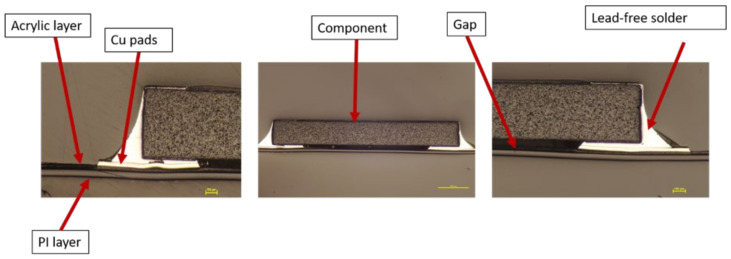
Cross-section images for flat surface.

**Figure 21:**
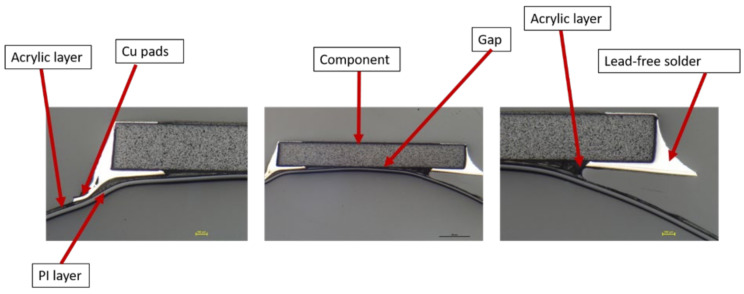
Cross-section images for curved surface.

Moreover, a visual examination was conducted to verify that the solder connections between the components and the copper pad were intact. The cross-section images were obtained in two-component positions: on a flat surface and on a curved slightly formed surface. Figure 20 illustrates a component constructed on a flat surface, whereas Figure 21 depicts a component assembled on a curved surface.

Cross-section images of both positions, curved and flat surfaces, demonstrate the strong connection formed by assembled components and copper tracks when lead-free solder is used. Even in the case when the component is near the curvature’s location, the solder is attached to the acrylic adhesive layer. These images demonstrate our work’s novelty on why solder joints are preferred in the over-molding process when using acrylic-based flexible foils. Additionally, it may be used in curvatures with a small radius, such as the 4 mm in our case.

##### Measurements on Samples Assembled with Conductive Adhesive

The resistance of the assembled component was tested before and after over-molding and calculated again using Eq.1. As indicated before in the shear force section (Table 3), components bonded with conductive glue yielded lower shear force at break than soldered components. This affected the electrical measurements in some way.

**Figure 22:**
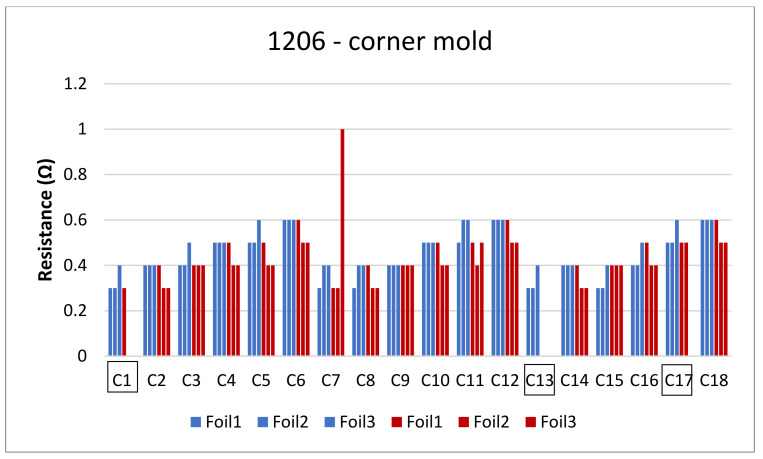
Resistance measurements for 1206 resistors molded in corner mold.

Figure 22 depicts the resistance for the foils assembled with 1206-size components. Some components had very high resistance values that led to an open loop (OL) reading on the multimeter. Such reading means that resistance is opposition to the free flow of current within a circuit and the higher the resistance, the harder it is for the current to flow from one point to another. Other components were out of place after over-molding, and, for both cases, their resistance was eliminated from the calculations in order to have more accurate data as is the case for C1, C13, and C17 in Figure 22.

**Figure 23:**
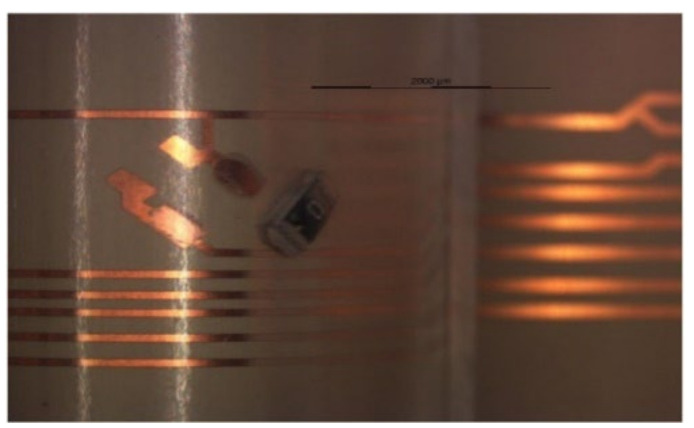
An out-of-place component at 2000 µm.

After over-molding, components number 13 (for two foils) had no data after over-molding. Therefore, we investigated the foils and found that the three components of C13 were out of place after over-molding as depicted in Figure 23.

**Figure 24:**
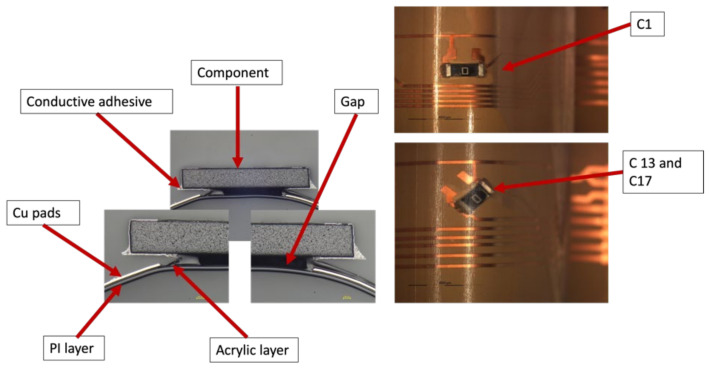
Cross-sectional images at 100 µm for C1, C13, and C17 components (**Left**). Top view of the component at 3000 µm (**Right**).

However, in the case of C1, the components were on the foil but were not conducting. Therefore, cross-section images were taken to check the conductive adhesive connection reliability between copper pads and components’ conductive adhesive joints, as depicted in Figure 24. This figure shows how a curved surface may disconnect the copper and the acrylic layer from the conductive adhesive. The same failure was also detected for C17 and C13 on foil 3.

**Figure 25:**
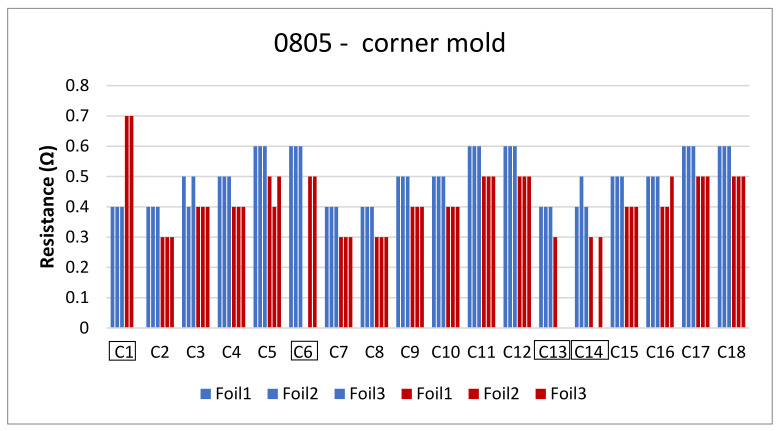
Resistance measurements for 0805 resistors molded in corner mold.

On the other hand, Figure 25 depicts the resistance measurements for 0805-size components. Four components had OL readings after over-molding, these components are C1, C6, C13, and C14.

**Figure 26:**
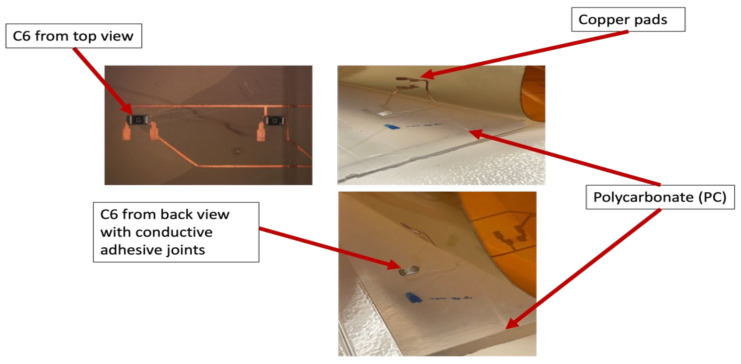
Poor adhesion between PI-Cu and PC.

Regarding components C1, C13, and C14, the same concept of the weak bond between the conductive adhesive and the joints apply as presented in Figure 24. However, for C6, the conductive adhesive joints were on the component, but due to poor adhesion between the PC and PI-Cu-based foil, the copper traces were delaminated from the conductive adhesive joints and stayed on the components, thus, the connection was broken as depicted in Figure 26.

**Figure 27:**
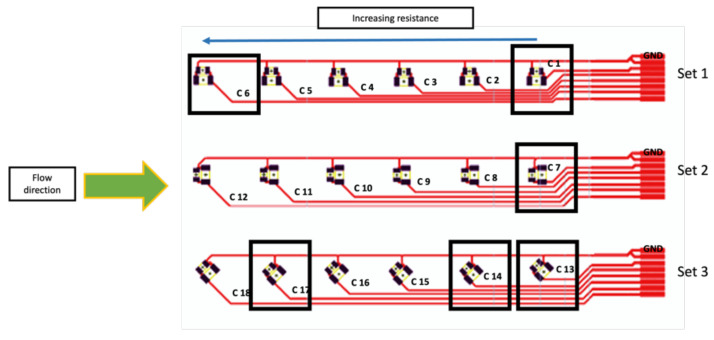
Typical failure locations for the corner mold.

Generally, components cannot be incorporated or present in curvatures as part of the in-mold technology design requirements. However, the components in the lead-free solder-based foils were functional after over-molding despite the curvature. Moreover, according to our tests, the use of the lead-free solder on copper pads gives a stronger connection between the component and the pad as proven in our shear tests. We can conclude that a corner shape mold and weaker bonds between components and the used foil could lead to several failures. Figure 27 depicts the failure locations. Components C1, C7, C14, and C13 could have a high change in resistance due to their position in the curved area. Moreover, components C6 and C17 which are close to the flow entry point may become displaced or out of place due to the polymer flow.

**Figure 28:**
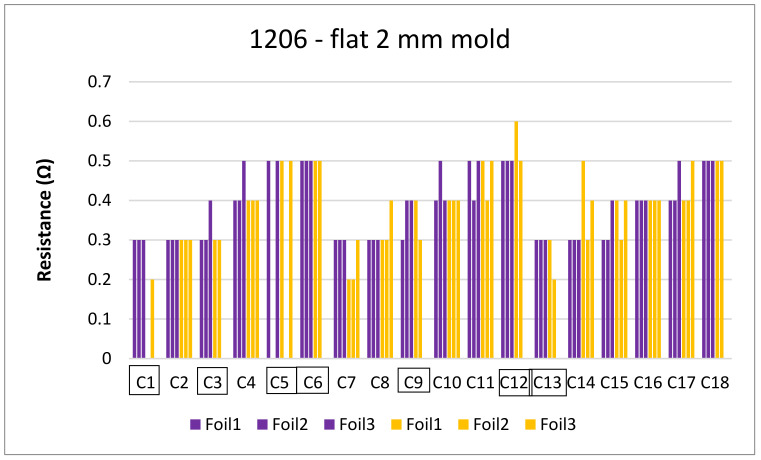
Resistance measurements for 1206 resistors molded in the 2 mm flat mold.

Regarding the 2 mm flat mold, Figure 28 depicts the resistance measurements for 1206 resistors before (purple bars) and after (yellow bars) over-molding. In foil 2, component C5 was not assembled in a reliable way due to manual assembly and, therefore, there is no data present for it before and after over-molding.

**Figure 29:**
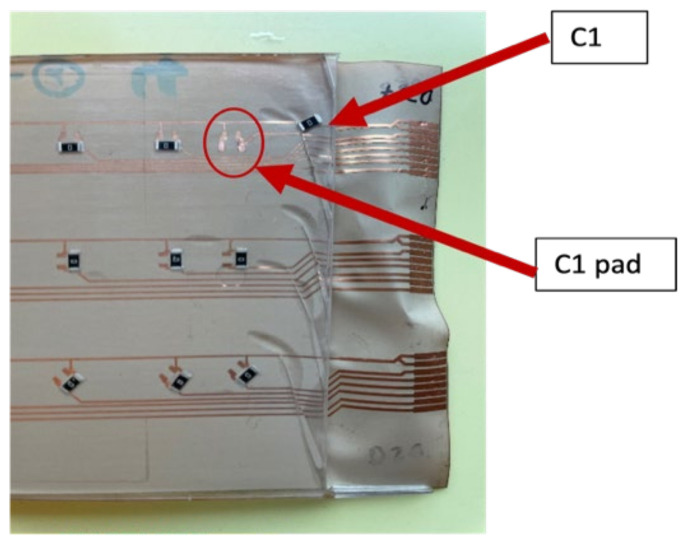
An out-of-place component.

**Figure 30:**
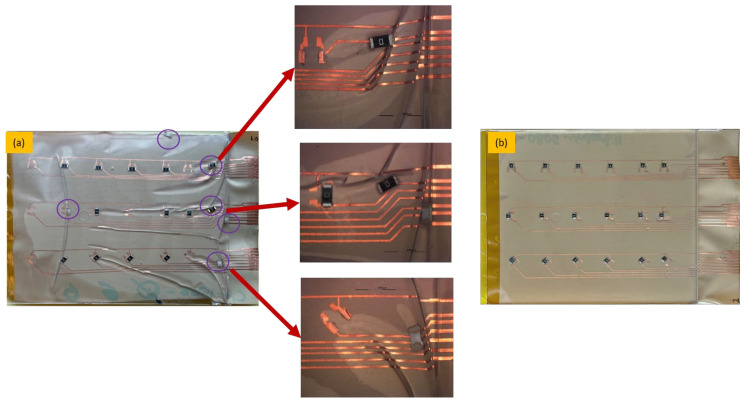
Bad over-molding with misplaced foil, removed components and wrinkles (**a**), good over-molded foil with all components and without wrinkles (**b**).

In this group of foils, we had failures for foil 1 and foil 3. For foil 1, C1 was out of place after over-molding as depicted in Figure 29. On the other hand, for foil 3, many components were out of place. The main reason for such an observation is the improper alignment of the foil within the mold, resulting in a wrinkled foil with removed components as presented in Figure 30.

Most foils that had problems in terms of detached components and wrinkled foil appearance were over-molded at low-melt temperatures of 240 °C and 260 °C. At melt temperatures of 280 °C and 300 °C, no detachment was observed on the foils using CA regardless of the used injection speed or mold thickness.

**Figure 31:**
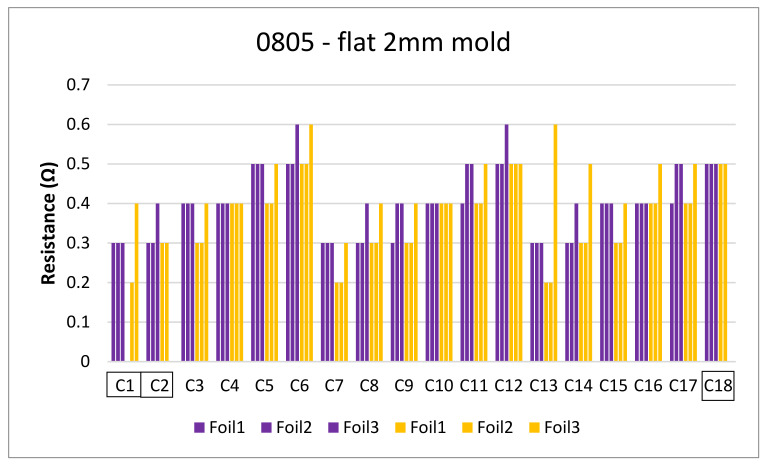
Resistance measurements for 0805 resistors molded in the 2 mm flat mold.

**Figure 32:**
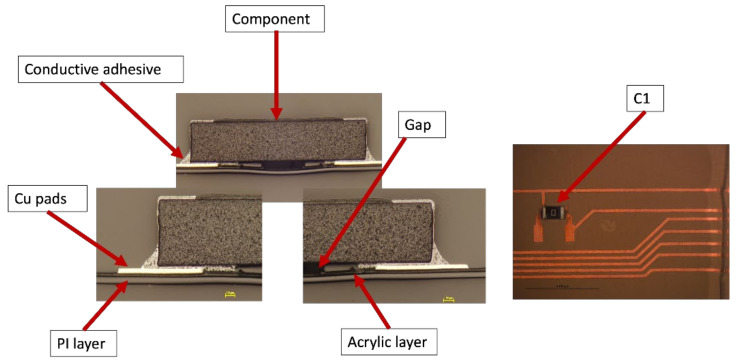
Cross-sectional images at 100 µm for C1 component (**Left**). Top view of the component at 3000 µm (**Right**).

Figure 31 shows the resistance for 0805 components in a 2 mm flat mold. As depicted, one of the C1 components gave an open loop reading and was still on the foil. Therefore, cross-section images were taken to check the conductive adhesive joints on a flat surface as depicted in Figure 32.

**Figure 33:**
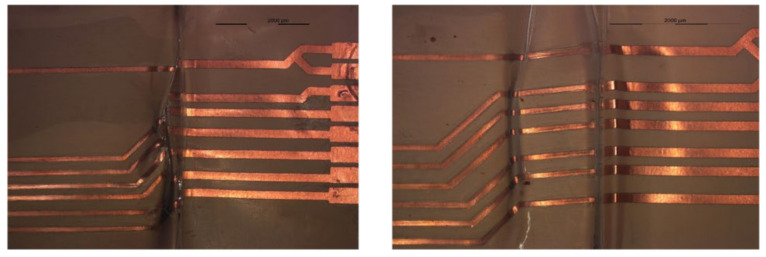
Compressed copper tracks at 2000 µm magnification.

Additionally, as indicated before in Section 2.3, the detachable inserts were added to protect the contacts and to facilitate the resistance measurements after over-molding. However, the films occasionally developed wrinkles as a result of over-molding during the transition to the insert and some of the foils had compressed copper tracks, as illustrated in Figure 33. This wrinkling effect caused a discontinuity in the copper tracks and, therefore, some components also had very high resistance as in the case of components C2 and C18.

In general, such wrinkle failure may also occur in solder-based foils as well since it is a failure due to clamping the foils into the mold using the inserts, not the material of assembly used.

**Figure 34:**
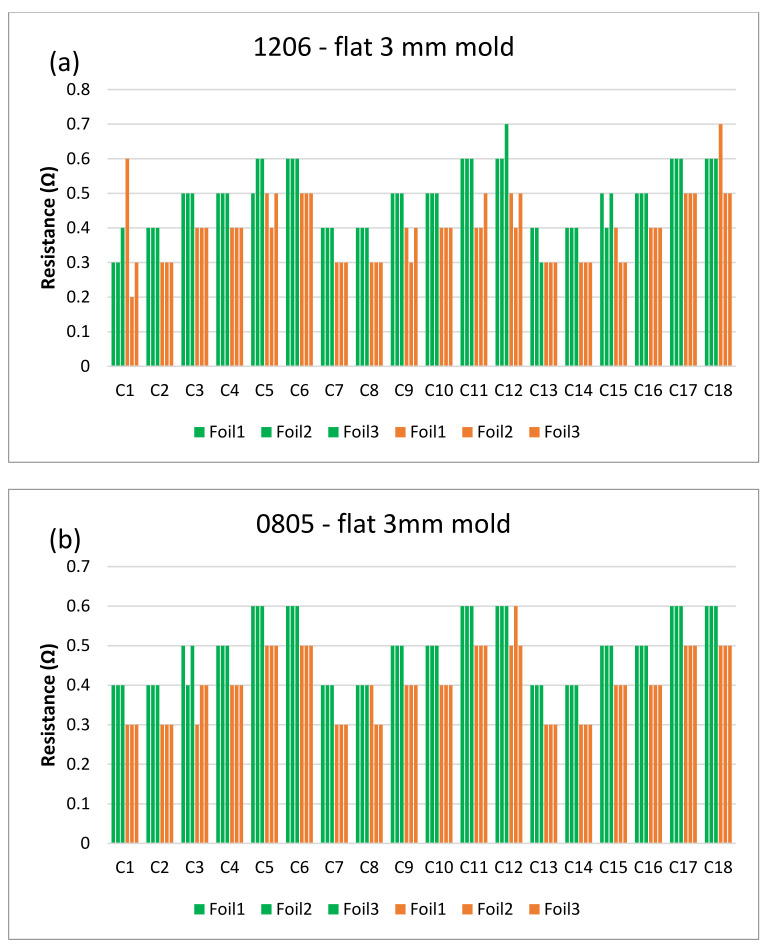
Resistance measurements for 1206 resistors (**a**) and 0805 resistors (**b**) molded in the 3 mm flat mold.

Finally, the resistance measurements were taken for the 3 foils over-molded in the 3 mm plate mold, green bars represent resistance before over-molding tests and orange bars represent after over-molding as depicted in Figure 34.

With the used settings, foils over-molded in the 3 mm mold showed fewer failures compared to those in the 2 mm and the corner molds. This is due to the lower prevailing shear stresses and pressure during filling. Moreover, the components assembled using conductive adhesive varied significantly from those assembled with solder. This could be because of the nature of the conductive adhesive material that makes it difficult to manually apply it in equal joints during the assembly step.

#### 3.2.2. Measurements on PET-Cu Foils

##### Measurements on Samples Assembled with Low-Temperature Solder

**Figure 35:**
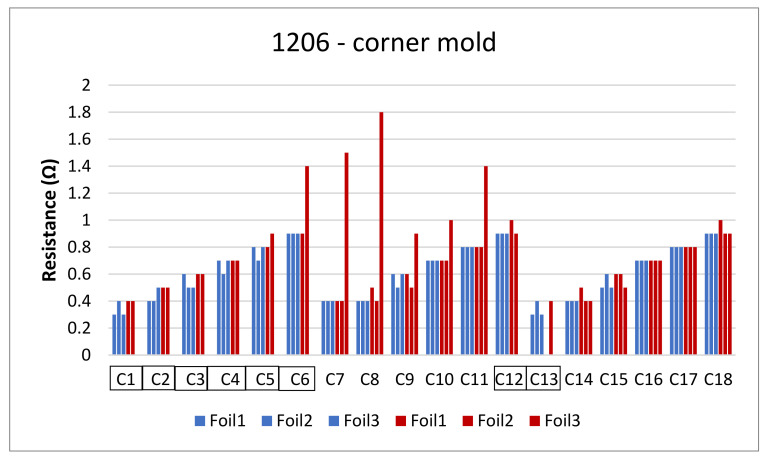
Resistance measurements for the corner mold.

In this section, we used only 1206 components for assembly. This is because, according to our study, component size is independent of the electrical measurements and also because they are easier when manually soldered. Figure 35 depicts the resistance values for 1206 resistors over-molded in the corner mold taken before (blue bars) and after (red bars) over-molding. Components bonded with low-melt solder yielded less shear force at break lower than those bonded with solder and higher than those using conductive adhesive as discussed in Section 3.1.

**Figure 36:**
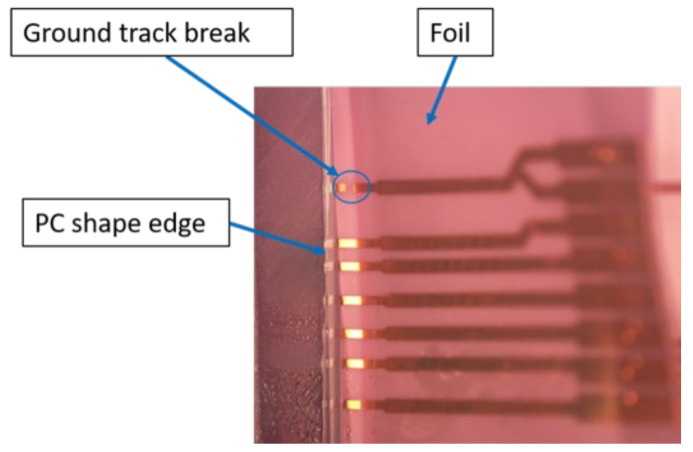
Ground track break in foil 3.

Foil 3 had a ground break as depicted in Figure 36, affecting the measurements between C1-C6. Some components were out-of-place after over-molding like in the case of C12 and C13.

**Figure 37:**
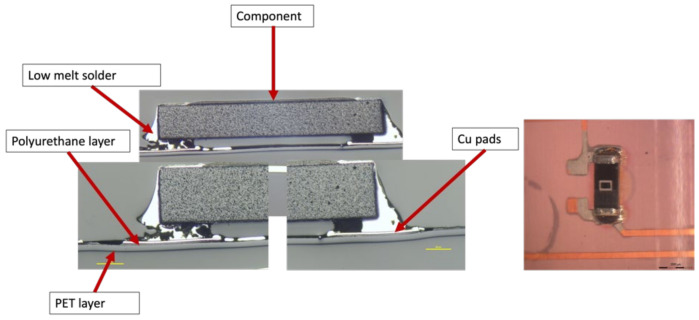
Cross-sectional images at 500 µm for component C7 (**Left**). Top view of the component at 2000 µm (**Right**).

Cross-section images were taken for one of the components, C7, which is in the curved area to check the connection reliability for the low-melt solder on such a surface as depicted in Figure 37. These images show that the low-melt solder was not fully connected to the copper pad, yet component C7 was functional after over-molding.

**Figure 38:**
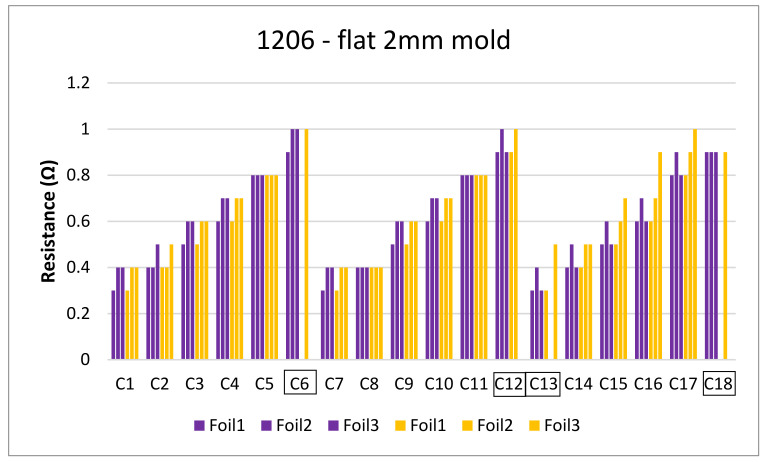
Resistance measurements for flat mold-2 mm.

Figure 38 displays the resistance measurements of the components over-molded in the 2 mm plate mold before (purple bars) and after (yellow bars).

**Figure 39:**
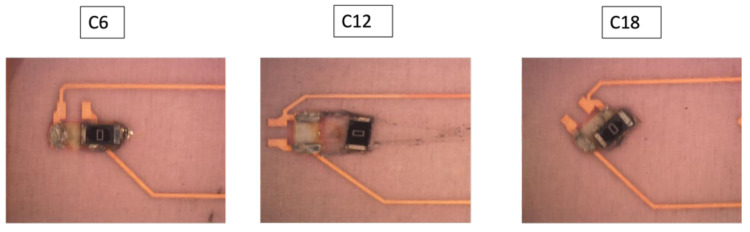
Out-of-place components.

Some components C6, C12, C13, and C18 were not included in our measurements since they were out of place after over-molding as depicted in Figure 39.

**Figure 40:**
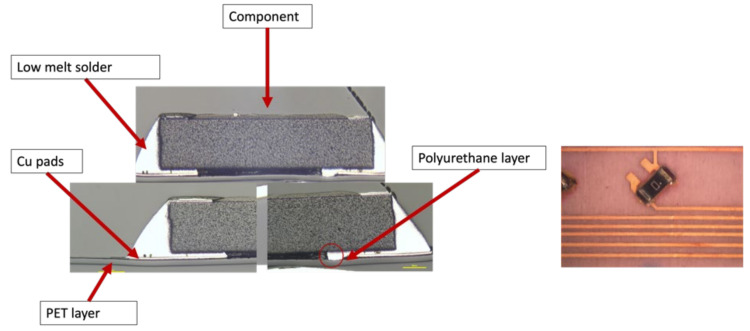
Cross-sectional images at 500 µm for component C13 (**Left**). Top view of the component at 2000 µm (**Right**).

Moreover, some components remained on the foil after over-molding but had high resistance measurements as was the case for C13. In this case, cross-sectional images were taken to assess the connection reliability between copper pads and low-melt solder joints, as depicted in Figure 40. This image shows a discontinuity in the copper and the PU layer.

**Figure 41:**
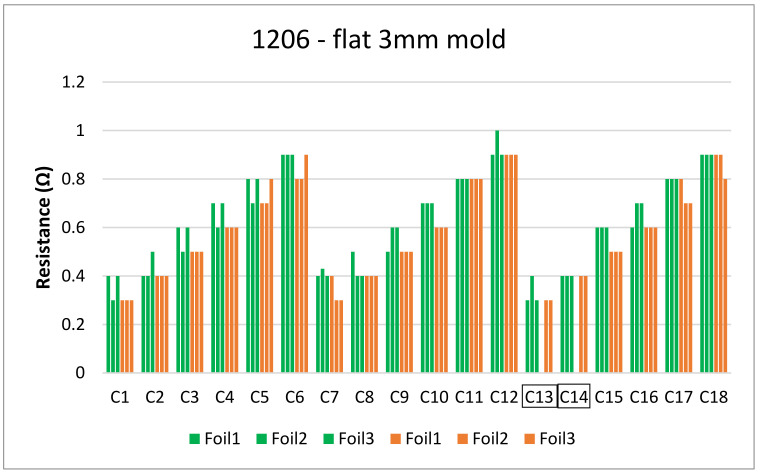
Resistance measurements for flat mold-3 mm for 1206 resistors.

As shown in Figure 41, measurements were taken in a flat mold with a thickness of 3 mm, with green bars representing testing before over-molding and orange bars representing after over-molding. Only one of each component, C13 and C14, were removed from our measurements since they led to very high resistance which caused an infinite resistance reading.

##### Measurements on Samples Assembled with Conductive Adhesive

**Figure 42:**
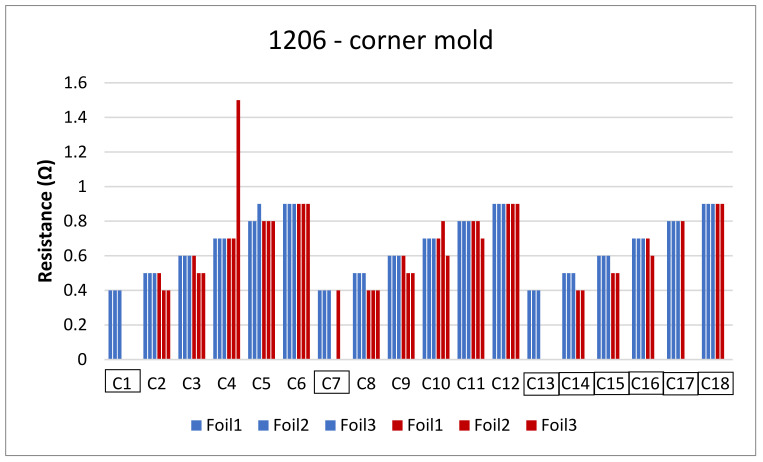
Resistance measurements for corner mold.

In the literature, low-temperature solder was the material used to assemble the electrical components on PET foils [33,34,35]. However, we considered using conductive adhesive since it also cures in low-temperature conditions at 120 °C and the degradation process of PET at such temperature is relatively slow [36]. Figure 42 depicts the resistance values in the corner mold. The change in resistance measurements was taken before (blue bars) and after (red bars) over-molding. All foils were over-molded using a high melt temperature of 300 °C. It was observed that these foils had many components that were detached after over-molding.

**Figure 43:**
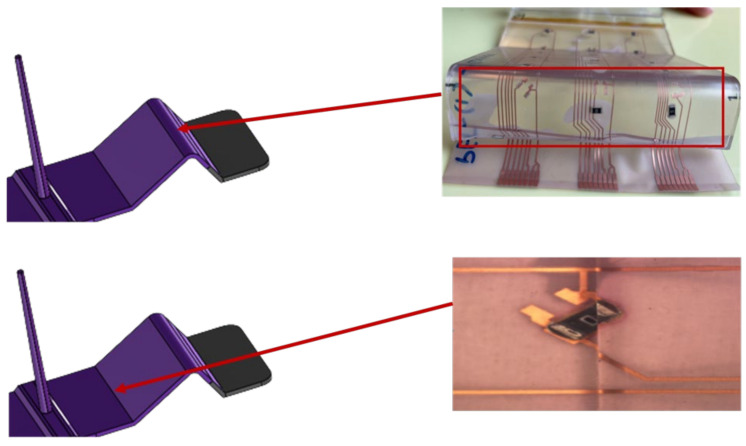
Corner mold critical areas.

Foils 1 and 2 had some detached components (C1, C7, C13, and C17) after over-molding because these components were molded in the corner mold but also in the critical areas as well as the weak bonds of CA as depicted in Figure 43.

**Figure 44:**
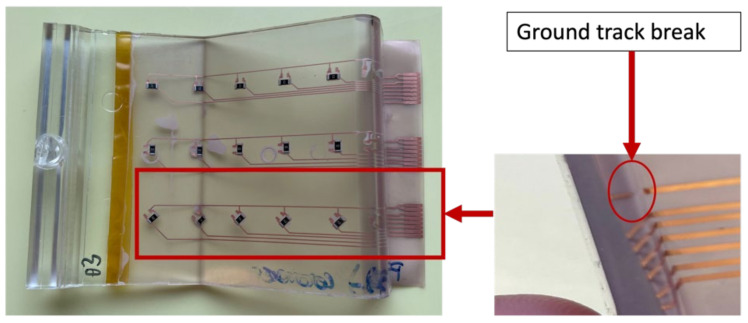
Ground track break in the corner mold in foil 3.

In addition to this, the ground track of foil 3 that connects C13-C18 was broken as depicted in Figure 44.

**Figure 45:**
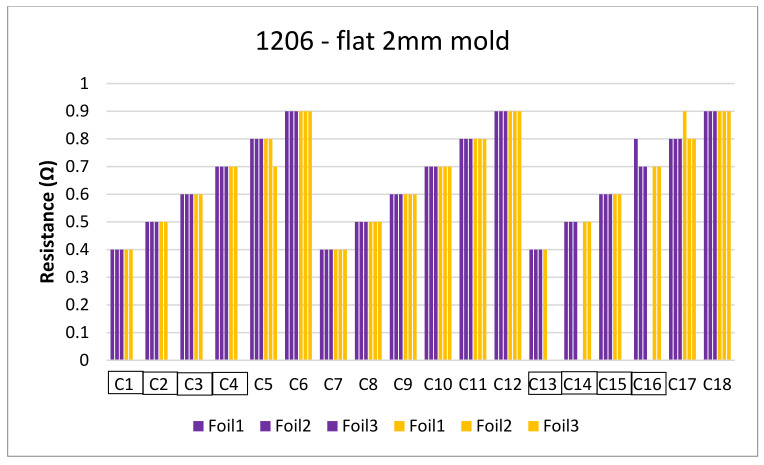
Resistance measurements for flat mold-2 mm for 1206 resistors.

**Figure 46:**
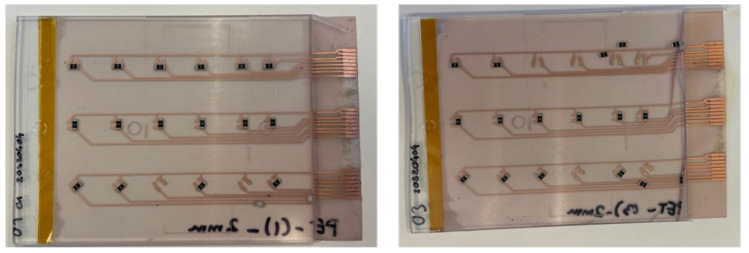
Example of detached components.

The resistance readings of the components over-molded in the 2 mm flat mold are depicted in Figure 45 before (purple bars) and after (yellow bars) and most of the components were detached after over-molding as depicted in Figure 46.

**Figure 47:**
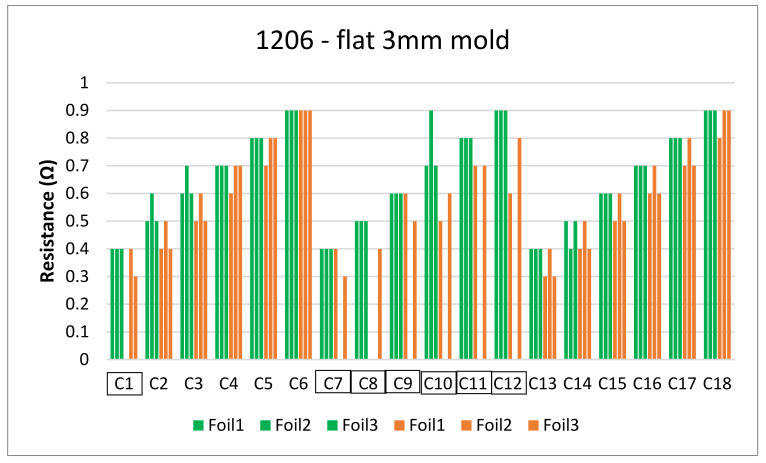
Resistance measurements for flat mold-3 mm for 1206 resistors.

Measurements were made in a flat mold with a thickness of 3 mm, as shown in Figure 47, where green bars denote testing before over-molding and orange bars denote measurements after over-molding. The resistivity of the majority of components had changed because of the break in the ground track in foil 2, which led to non-conducting components between C7–C12. Moreover, foil 1 had a detached component (C1).

## 4. Conclusion

This paper analyzed the use of different assembly materials on PI and PET copper-based foils. Regarding soldered components on PI-Cu foils, all resistors yielded 100% success after over-molding. In contrast, the majority of the components assembled using conductive adhesive on PI-Cu and PET-Cu foils as well as low-melt soldered components on PET-Cu foils were detached after over-molding. Moreover, components should be located in areas with lower deformation and fewer stress concentrations to prevent damage, particularly when assembled using conductive adhesive and low-melt solder. However, glob-top and underfill materials could be used and may protect the components in such regions. From our experiments, failures were detected more after the over-molding of components using conductive adhesive and low-melt solder. Such failures include foil misalignment, which led to removed components and non-functional components because of the conductive adhesive and low-melt solder weak bonds on copper tracks. A relatively high mold temperature of 100 °C was found to be beneficial for good adhesion with PI (acrylic adhesive layer) and PET foils (polyurethane adhesive layer) compared to a lower mold temperature of 80 °C. Generally, it seems that a lower melt temperature, a higher injection speed, and a lower mold thickness increase the stresses on the components during over-molding which, in turn, leads to larger resistance changes after over-molding. Additionally, the electrical measurements of the over-molded foils were unaffected using varied package sizes and assembly in different orientations.

## Figures and Tables

**Table 1:** Composition of different commercial copper cladding foils.

Foil Layers	FR9210 [27]	GTS5500 [28]
**Polymer layer**	50 µm PI layer	50 µm PET layer
**Adhesive layer**	25 µm acrylic-based adhesive layer	20 µm polyurethane-based adhesive layer
**Copper (Cu) layer**	35 µm	18 µm

**Table 2:** Listing of the over-molded parts for peel test.

No.	Foil	Mold Temperature (°C)	Residual Cooling Time (s)	Surface Treatment
**PI-I**	FR9120	80	50	-
**PI-II**	FR9120	100	50	-
**PI-III**	FR9120	100	50	-
**PI-IV**	FR9120	100	50	Corona
**PI-V**	FR9120	100	20	Oxygen plasma
**PI-VI**	FR9120	120	50	-
**PET-I**	GTS	80	50	-
**PET-II**	GTS	100	50	-
**PET-III**	GTS	100	50	Corona
**PET-IV**	GTS	100	50	Oxygen plasma

**Table 3:** Shear force for assembled components.

Shear Force and Standard Deviation	1206	0805
**Solder on PI foils**	62 ± 4 N	41 ± 5 N
**Low-melt solder on PET foils**	43 ± 3 N	31 ± 1 N
**CA on PI foils**	39 ± 8 N	29 ± 6 N

**Table 4:** Average resistance values of the assembled resistors before OVM.

Components	Resistance Values (*R_measured_*)					
**C1 to C6**	R1 = 0.2 Ω	R2 = 0.3 Ω	R3 = 0.3 Ω	R4 = 0.4 Ω	R5 = 0.4 Ω	R6 = 0.5 Ω

**Table 5:** Difference between *R_measured_* and *R_theoretical_*.

	Length between Resistor and Contact Pad	*R_measured_*	*R_theoretical_*
**R1**	32.5 mm	0.2 Ω	0.305 Ω
**R2**	41.24 mm	0.3 Ω	0.33 Ω
**R3**	54.78 mm	0.3 Ω	0.37 Ω
**R4**	68.45 mm	0.4 Ω	0.42 Ω
**R5**	82.3 mm	0.4 Ω	0.46 Ω
**R6**	97.4 mm	0.5 Ω	0.52 Ω

## Data Availability

Not applicable.

## References

[1] Pötsch G., Michaeli W. (2008). Injection Molding: An Introduction.

[2] Singh G., Verma A. (2017). A brief review on injection moulding manufacturing process. Mater. Today Proc..

[3] Teh N.J., Prosser S., Conway P.P., Palmer P.J., Kioul A. Embedding of electronics within thermoplastic polymers using injection moulding technique. Proceedings of the Twenty Sixth IEEE/CPMT International Electronics Manufacturing Technology Symposium (Cat. No.00CH37146).

[4] Bakr M., Bossuyt F., Vanfleteren J. (2022). The integration of electronic circuits in plastics using injection technologies: A literature review. Flex. Print. Electron..

[5] Alajoki T., Matti K., Markus T., Mikko H., Antti K., Kimmo K., Jukka-Tapani M., Janne A., Kari R. Hybrid in-mould integration for novel electrical and optical features in 3D plastic products. Proceedings of the 4th Electronic System-Integration Technology Conference.

[6] Rusanen O., Simula T., Niskala P., Lindholm V., Heikkinen M. Injection Molded Structural Electronics Brings Surfaces to Life. Proceedings of the 2019 22nd European Microelectronics and Packaging Conference & Exhibition (EMPC).

[7] Juntunen E., Ihme S., Huttunen A., Mäkinen J. R2R process for integrating LEDs on flexible substrate. Proceedings of the 2017 IMAPS Nordic Conference on Microelectronics Packaging (NordPac).

[8] Kololuoma T., Keränen M., Kurkela T., Happonen T., Korkalainen M., Kehusmaa M., Gomes L., Branco A., Ihme S., Pinheiro C. (2019). Adopting Hybrid Integrated Flexible Electronics in Products: Case—Personal Activity Meter. IEEE J. Electron Devices Soc..

[9] Bakr M., Bauwens P., Bossuyt F., Vanfleteren J., Chtioui I., Christiaens W. Solar cells integration in over-molded printed electronics. Proceedings of the 2020 IEEE 8th Electronics System-Integration Technology Conference (ESTC).

[10] Bakr M., Bossuyt F., Vanfleteren J., Su Y. (2020). Flexible Microsystems Using Over-molding Technology. Procedia Manuf..

[11] Wimmer A., Reichel H., Schmidt S. New standards for 3D-userinterfaces-manufactured by a Film Insert Molding process. Proceedings of the 2018 13th International Congress Molded Interconnect Devices (MID).

[12] Gbotemi O., Myllymäki S., Jantunen H., Juuti J., Ihme S., Kurkinen M., Majava V., Tuhkala M., Kemppainen J. (2020). Printed GNSS and Bluetooth Antennas Embedded on Flexible Low Loss Substrates for Wearable Applications. Prog. Electromagn. Res. M.

[13] Tuomikoski M., Ihme S., Huttunen A., Korkalainen M., Yrjänä S. Indoor air quality sensing indicators. Proceedings of the 2016 6th Electronic System-Integration Technology Conference (ESTC).

[14] Nguyen S., Perez C.J., Desimone M., Pastor J.M., Tomba J.P., Carellaa J.M. (2013). Adhesion control for injection overmolding of elastomeric propylene copolymers on polypropylene. Effects of block and random microstructures. Int. J. Adhes. Adhes..

[15] Stan F., Fetecau C. Experimental Investigation of the Adhesion Between Thermoplastic Polyurethane and Acrylonitrile-Butadiene-Styrene Substrate. Proceedings of the ASME 2014 International Manufacturing Science and Engineering Conference collocated with the JSME 2014 International Conference on Materials and Processing and the 42nd North American Manufacturing Research Conference. Volume 2: Processing.

[16] Ott C., Wolf M., Drummer D. (2020). Media-Tight Polymer-Polymer Assemblies By Means of Sintered Powder Layer in Assembly Injection Moulding. Procedia Manuf..

[17] Leong Y.W., Ishiaku U.S., Kotaki M., Hamada H., Yamaguchi S. (2006). Interfacial characteristics of film insert molded polycarbonate film/polycarbonate-acrylonitrile-butadiene-styrene substrate, part 1: Influence of substrate molecular weight and film thickness. Polym. Eng. Sci..

[18] Leong Y.W., Ishiaku U.S., Kotaki M., Hamada H., Yamaguchi S. (2005). Effect of crystallization and interface formation mechanism on mechanical properties of film-insert injection-molded poly(propylene) (PP) film/PP substrate. J. Appl. Polym. Sci..

[19] Chen S.C., Li H.M., Huang S.T., Wang Y.C. (2010). Effect of decoration film on mold surface temperature during in-mold decoration injection molding process. Int. Commun. Heat Mass Transf..

[20] Chen H.L., Chen S.C., Liao W.H., Chien R.D., Lin Y.T. (2013). Effects of insert film on asymmetric mold temperature and associated part warpage during in-mold decoration injection molding of PP parts. Int. Commun. Heat Mass Transf..

[21] Baldan A. (2012). Adhesion phenomena in bonded joints. Int. J. Adhes. Adhes..

[22] Awaja F., Gilbert M., Kelly G., Fox B., Pigram P.J. (2009). Adhesion of polymers. Prog. Polym. Sci..

[23] Creton C., Kramer E.J., Brown H.R., Hui C.Y. (2001). Adhesion and Fracture of Interfaces Between Immiscible Polymers: From the Molecular to the Continuum Scal. Molecular Simulation Fracture Gel Theory.

[24] Cole P.J., Cook R.F., Macosko C.W. (2003). Adhesion between immiscible polymers correlated with interfacial entanglements. Macromolecules.

[25] https://www.campusplastics.com/campus/de/datasheet/Makrolon%C2%AE+2805/Covestro+Deutschland+AG/22/7541f4aa.

[26] LeGrand D.G., Bendler J.T. (2000). Plastics engineering, Bd. 56. Handbook of Polycarbonate Science and Technology.

[27] https://www.dupont.com/content/dam/dupont/amer/us/en/products/ei-transformation/documents/EI-10113-Pyralux-FR-CCL-Data-Sheet.pdf.

[28] https://www.gtsflexible.com/product-table/?tx_gtsproducts_products%5Baction%5D=list&tx_gtsproducts_products%5Bcontroller%5D=Products&cHash=b0ee19a0fdbc58db79b019bffba1c77c#productList.

[29] https://www.professionalplastics.com/professionalplastics/content/AcryliteFFDataSheet.pdf.

[30] Bath J. (2007). Lead-Free Soldering.

[31] https://www.henkel-adhesives.com/be/en/product/electrically-conductiveadhesives/loctite_ablestikce3103wlv.html.

[32] https://interflux.com/en/product/DP-5600.

[33] Wimmer A., Reichel H., Rauch B., Schramm R., Hörber J., Hä:ßler B. Manufacturing of sandwich structures for the integration of electronics in in mold labelling components. Proceedings of the 2016 12th International Congress Molded Interconnect Devices (MID).

[34] Alajoki T., Koponen M., Juntunen E., Petaja J., Heikkinen M., Ollila J., Sitomaniemi A., Kosonen T., Aikio J., Makinen J.T. In-mould integration of electronics into mechanics and reliability of overmoulded electronic and optoelectronic components. Proceedings of the 2009 European Microelectronics and Packaging Conference.

[35] Koponen M., Alajoki T., Kosonen T., Petäjä J., Heikkinen M., Vuorinen T., Mäkinen J.-T. (2008). Adhesion of Flexible Printed Circuit Substrate to Overmoulded Polymer and Characterization of Overmoulded Electronic Components. IMAPS Nordic Annual Conference Denmark 2008.

[36] Panowicz R., Konarzewski M., Durejko T., Szala M., Łazińska M., Czerwińska M., Prasuła P. (2021). Properties of Polyethylene Terephthalate (PET) after Thermo-Oxidative Aging. Materials.

